# The Prevalence of the Potential Drug-Drug Interactions Involving Anticancer Drugs in China: A Retrospective Study

**Published:** 2019-03

**Authors:** Weilan WANG, Bingkun XIAO, Ziqi LIU, Dongxiao WANG, Man ZHU

**Affiliations:** 1. Department of Pharmacy, General Hospital of People’s Liberation Army (PLA), Beijing, 100853, China; 2. Department of Pharmacochemistry, Institute of Radiation Medicine, Beijing, 100850, China; 3. Department of Pharmacy, General Hospital of People’s Liberation Army (PLA) Rocket Force, Beijing, 100088, China

**Keywords:** Anticancer drugs, Drug-drug interactions, Prevalence, China

## Abstract

**Background:**

To survey the prevalence of potential drug-drug interactions (DDIs) between anticancer drugs and non-anticancer drugs and evaluate the risk factors associated with these drug-drug interactions in China.

**Methods:**

All discharged patients in the Department of Oncology were collected from Jun to Dec in 2016 with the Hospital Information System of the Chinese people’s Liberation Army General Hospital. Drugs were screened for interactions by Micromedex solutions database. Descriptive statistics were generated and logistic regression was used to identify the associated factors.

**Results:**

Among 6578 eligible patients, 1979 potential drug interactions were found in 1830 patients (27.82%). The most common drug-drug interaction was cisplatin and furosemide. Erlotinib was most likely to interact with various non-anticancer drugs. Most interactions were classified as pharmacodynamics (71.60%), major severity (97.02%) and were supported by fair documentation evidence (86.21%). In multivariate analysis, increasing number of medications, lung cancer and patients with stage IV had a higher risk for potential drug-drug interactions.

**Conclusion:**

Potential drug-drug interactions between antineoplastic drugs and non-antineoplastic drugs occur frequently in cancer patients of Chinese hospitals. Doctors should fully consider potential risk associated with DDIs. Further research should be performed to evaluate real clinical significance of these drug-drug interactions.

## Introduction

Drug-drug interactions (DDIs) are defined as a medication interferes with pharmacokinetic, pharmacodynamic, or pharmaceutical properties of another drug, resulting in an altered net effect of one or both drugs (1). DDIs could be a significant cause of morbidity and mortality because they may result in enhancement of drug toxicity and decrease in therapeutic effects of drugs (2–4). DDIs are of particular concern in oncology because anticancer drugs usually have a narrow therapeutic index and small changes in cytotoxic activity due to a DDI can have serious consequences (5). For example, concurrent use of nonsteroidal anti-inflammatory drugs (NSAIDs) and methotrexate may result in an increased risk of methotrexate toxicity, and fatal cases have been reported (6, 7). Dual CYP3A4 and CYP1A2 inhibitor ciprofloxacin may result in increased erlotinib exposure and even lead to death (8). Patients receiving anti-cancer therapy are particularly vulnerable to DDIs because they often take numerous medications concurrently to manage their malignancy, toxicities, cancer-associated syndromes and other co-morbid illnesses. These multiple medications can increase the risk of DDIs markedly (9, 10).

However, very limited data are available for potential drug interactions associated with anti-cancer therapy. In Belgium, forty-one potential interactions involving an anticancer agent and considered to be clinically significant were identified among 25% of patients (11). Overall, ∼5% of patients taking oral anticancer agents in Singapore were exposed to≥1 potentially interacting drug combination (10). An observational study using medical records and autopsy analysis showed that 4% of deaths among cancer patients were caused by chemotherapy itself, and serious drug-drug interactions were sometimes suspected (12).

To date, there was no relevant research data about DDIs involving anticancer agents in China. The Chinese people’s liberation army general hospital is the largest comprehensive hospital in China, owning more than 4400 beds in all and more than 300 beds in oncology. Considering combination between antineoplastic drugs and antineoplastic drugs sometimes are unavoidable as standard treatment such as concurrent use of cisplatin and paclitaxel despite enhancement of drug toxicity for DDIs, the main purpose of this study was to survey the prevalence of potential DDIs between antineoplastic drugs and nonantineoplastic drugs and evaluate the risk factors associated with these DDIs in the largest comprehensive hospital in China.

## Materials and Methods

### Study subjects

All discharged patients in the Department of Oncology were collected from Jun to Dec in 2016 with the Hospital Information System of the Chinese people’s Liberation Army General Hospital. The study protocol was approved by the Medical Ethics Committee of the hospital.

### Experimental protocol

The inclusion criteria were: patients who received at least one anti-cancer drug and one nonanticancer drug simultaneously during oncology department stay were considered eligible. Anti-cancer agents include all traditional anti-tumor agents and molecule-targeted agents, and administration route was defined as intravenous and oral administration route.

For each patient included, we collected the following data from the electronic medical record: age, sex, medications administered concurrently for anti-cancer drugs and non-anti-cancer drugs, type of cancer, tumor stage, and number of medication orders. Micromedex solutions database was utilized to identify potential interactions administered simultaneously between anti-cancer drugs and non-anti-cancer drugs because it was recognized as one of the most useful resources for identifying potential clinical effects (13). Micromedex classifies DDIs into 5 categories of severity: contraindicated, major, moderate, minor, and unknown. For this study, only contraindicated, major and moderate were selected because interactions of minor severity lack clinical significance. Definitions for severity and documentation of DDIs by Micromedex are shown in Table 1. Regarding mechanism of action, DDIs were classified as either pharmacokinetics or pharmacodynamics. Pharmaceutical interactions were not investigated because they were beyond the scope of our study. Tumor stages are classified in the study as stage IV and prior to stage IV because patients with stage IV face more complex physical, psychological, social, and spiritual consequences of disease and more treatment including disease-directed therapy, symptom management, and attention to quality of life compared with prior to stage IV (14–17).

**Table 1:** Micromedex classification criteria for DDIs

***Classification***	***Definition***
Severity	
Contraindicated	The drugs are contraindicated for concurrent use
Major	The interaction may be life-threatening and/or require medical intervention to minimize or prevent serious adverse effects
Moderate	The interaction may result in exacerbation of the patient’s condition and/or require an alteration in therapy
Minor	The interaction would have limited clinical effects
Documentation	
Excellent	Controlled studies have clearly established the existence of the interaction
Good	Documentation strongly suggests the interaction exists, but well-controlled studies are lacking
Fair	Available documentation is poor, but pharmacologic considerations lead clinicians to suspect the interaction exists; or, documentation is good for a pharmacologically similar drug.
Unknown	Unknown

### Statistical analysis

We used descriptive statistics to summarize patient and potential drug interactions characteristics. Continuous variables were reported as mean±standard deviation (SD). The data were analyzed using independent sample t-test and chi-square test to compare the characteristic differences including age, sex, type of cancer, tumor stage and number of medication orders between patients with and without DDIs. Multiple logistic regression analysis was used to identify the factors from the statistically significant single factors. Data were analyzed using statistical software SPSS version 13.0 (Chicago, IL, USA) with test level α=0.05. *P*-values less than 0.05 were considered statistically significant.

## Results

From Jun to Dec in 2016, 6578 patients who received at least one anti-cancer drug and one non-anti-cancer drug simultaneously were identified. The characteristics of the 6578 eligible patients were summarized in Table 2.

**Table 2:** Patient characteristics (n=6578)

***Characteristics***	***N***	***%***
Sex		
Male	3134	47.64
Female	3444	52.36
Age		
Mean±SD	54.82±11.16	
Range	12–86	
Number of medication orders		
Mean±SD	13.38±6.83	
Range	2–49	
Tumor stage		
Earlier than IV	2431	36.96
IV	4147	63.04
Cancer type		
Lung	1664	25.30
Breast	1408	21.40
Gastric	900	13.68
Intestinal	1273	19.35
Others ^*^	1333	20.26

* Others include pancreatic cancer, esophageal cancer, liver cancer, ovarian cancer, head and neck cancer, mesothelioma, melanoma, testicular cancer, neuroendocrine tumors, cholangiocarcinoma, adrenal cortical carcinoma, fibrous histiocytoma, prostate cancer, cervical cancer, trophoblastic tumor

The mean±SD age was 55.10±11.16 yr (range,12–86). More than half of the patients (52.36%) were females. Most patients (63.04%) were diagnosed with stage IV tumor. Lung cancer (25.30%), breast cancer (21.40%), intestinal cancer (19.35%) and gastric cancer (13.68%) were the most common cancer type. And the mean±SD number of medication orders per patient was 13.38±6.83 (range, 2–49).

Among 6578 eligible patients, 1979 potential drug interactions were found in 1830 patients (27.82%). One DDI was found in 1681 patients (91.86%), 2 in 149 patients (8.14%). 1867(94.34%) DDIs were identified in traditional anti-tumor agents and 112 (5.66%) DDIs were in molecule- targeted drugs. The severity of majority of DDIs was classified as major (97.02%) and only 2.98% as moderate. DDIs involving contra-indication were not found in the study. Documentation evidence of DDIs was classified as fair (86.21%), good (13.34%) and excellent (0.45%). Totally, 562 (28.40%) DDIs were pharmacokinetic and 1417(71.60%) DDIs were pharmacodynamic in pharmacological mechanism (Table 3). The most common drug-drug interactions are summarized in Table 4.

**Table 3:** Characteristics of potential drug interactions

***Characteristics***	***N***	***%***
Number of enrolled patients	6578	
Number of patients with ≥1potential drug interaction	1830	27.82
Number of DDIs	1979	
Drug subclass of DDIs		
Traditional anti-tumor drugs	1867	94.34
Molecule-targeted drugs	112	5.66
Severity of DDIs		
Major	1920	97.02
Moderate	59	2.98
Documentation level of DDIs		
Excellent	9	0.45
Good	264	13.34
Fair	1706	86.21
Pharmacological mechanism of DDIs		
Pharmacokinetic	562	28.40
Pharmacodynamic	1417	71.60

**Table 4:** The most common drug-drug interactions in this study population (n=1979)

***Drug-drug interaction***	***Probable effect***	***Number of DDIs***	***Severity***	***Documentation level***
Cisplatin Furosemide	Concurrent use may result in additive ototoxicity and/or nephrotoxicity	1379	Major	Fair
Pemetrexed NSAIDs	NSAIDs may increase pemetrexed toxicity	198	Major	Fair
Fluorouracil Cimetidine	Cimetidine may increase an increased risk of fluorouracil toxicity	168	Major	Good
Erlotinib				
PPI	PPI may decrease absorption of erlotinib	44	Major	Fair
Carbamazepine	Carbamazepine may result in decreased erlotinib exposure and potential loss of efficacy	11	Major	Fair
Rifampicin	Rifampicin may result in decreased erlotinib exposure and potential loss of efficacy.	3	Major	Fair
Warfarin	Concurrent use may result in an increased risk of bleeding	2	Major	Fair
Epirubicin Cimetidine	Cimetidine may result in an increased risk of epirubicin toxicity	48	Moderate	Good
Gefitinib				
PPI	PPI may decrease exposure of gefitinib	12	Major	Fair
Warfarin	Concurrent use may result increase risk of bleeding	7	Moderate	Excellent
Paclitaxel Carbamazepine	Carbamazepine may result in decreased exposure of paclitaxel	18	Major	Fair
Methotrexate	PPI may increase concentration of methotrexate and its metabolite	18	Major	Good
PPI				
Sunitinib	Concurrent use of sunitinb and ondansetron/levofloxacin/moxifloxacin may result in an increased risk of QT interval prolongation			
Ondansetron		6	Major	Fair
Levofloxacin		5	Major	Fair
Moxifloxacin		1	Major	Fair
Lapatinib				
Carbamazepine	Carbamazepine/ dexamethasone may decrease lapatinib exposure or plasma concentrations	2	Major	Excellent
Dexamethasone		6	Major	Fair
Sorafenib Dexamethasone	Dexamethasone may decrease sorafenib concentrations	4	Moderate	Fair
Ondansetron	Concurrent use may result in an increased risk of QT interval prolongation	3	Major	Fair

Overall, 1379 out of1979 (69.68%) DDI was cisplatin and furosemide, accounting for the largest percentage of DDIs. Moreover, erlotinib was most likely to interact with various nonanticancer drugs including PPI, rifampicin, warfarin, and carbamazepine.

Table 5 shows the characteristics of patients with and without DDIs. Patients with DDIs were more likely to be male, increasing number of medications, cancer type, and patients with stage IV (all, *P*<0.001). No significant differences were found in age (*P*>0.05). In multivariable analysis, increasing number of medications (odds ratio [OR] =1.09, 95% CI 1.08–1.11), cancer type (OR for lung vs. gastric tumors =4.59, 95% CI 3.79–5.56, other types of cancer vs. gastric tumors =2.03, 95%CI 1.66–2.48) and patients with stage IV (OR=1.52, 95%CI 1.32–1.76) remained significant associated with potential drug interactions. However, no significant differences were found in sex. Results of the multiple logistic regression analysis are presented in Table 6.

**Table 5:** Characteristics of patients with and without DDIs (n=6578)

***Characteristics***	***Patients with DDIs*** ***(n=1830)***	***Patients without DDIs*** ***(n=4748)***	***P***
Sex			<0.001
Male, n (%)	1153(63.01%)	1981(41.72%)	
Female, n (%)	677(36.99%)	2767(58.28%)	
Age			>0.05
Mean±SD	55.48±11.81	54.95±10.89	
Range	12–86	13–85	
Number of medications	<0.001		
Mean±SD	16±6.39	12±6.51	
Range	3–58	2–49	
Cancer type			<0.001
Lung	935(51.09%)	729(15.35%)	
Breast	134(7.32%)	1274(26.83%)	
Gastric	204(11.15%)	696(14.66%)	
Intestinal	18(0.98%)	1255(26.43%)	
Others ^*^	794(16.72%)	539(29.45%)	
Tumor stage			<0.001
Earlier than IV	476(26.01%)	1955(41.18%)	
IV	1354(73.99%)	2793(58.82%)	

* Others include pancreatic cancer, esophageal cancer, liver cancer, ovarian cance, head and neck cancer, mesothelioma, melanoma, testicular cancer, neuroendocrine tumors, cholangiocarcinoma, adrenal cortical carcinoma, fibrous histiocytoma, prostate cancer, cervical cancer, trophoblastic tumor

**Table 6:** Multivariable analysis for factors associated with DDIs

***Variable***	***Odds Ratio(95% CI)***	***P***
Sex	1.11(0.96–1.27)	>0.05
Number of medications	1.09(1.08–1.11)	<0.001
Cancer type		
Gastric	Referent	<0.001
Lung	4.59(3.79–5.56)	
Breast	0.73 (0.56–0.96)	
Intestinal	0.05 (0.03–0.09)	
Others ^*^	2.03(1.66–2.48)	
Tumor stage	1.52 (1.32–1.76)	<0.001

* Others include pancreatic cancer, esophageal cancer, liver cancer, ovarian cancer, head and neck cancer, mesothelioma, melanoma, testicular cancer, neuroendocrine tumors, cholangiocarcinoma, adrenal cortical carcinoma, fibrous histiocytoma, prostate cancer, cervical cancer, trophoblastic tumor

## Discussion

Overall, 1830 out of 6578 (27.82%) patients receiving antineoplastic therapy are exposed to at least one potential DDI. The prevalence observed in the study was comparable to the study of Belgium, 25% of patients were exposed to clinically significant DDIs (11). Considering high risk associated with DDIs in cancer patients, and almost all DDIs (97.02%) were classified as major, more attention should be paid by oncologists. Among the identified DDIs, the largest number of drug-drug interaction was cisplatin and furosemide. Furosemide is commonly used to mitigate nephrotoxicity of cisplatin. However, the protective effect of furosemide against nephrotoxicity has not been confirmed (18). In fact, furosemide enhanced nephrotoxicity of cisplatin (19–22). Twenty-six mg median dose of furosemide was associated with cisplatin nephrotoxicity and an explanation is that furosemide may have a direct toxic effect on the kidney (19). Furosemide-induced GFR reduction (20). High-dose furosemide leads to proximal tubular necrosis and its use with cisplatin may aggravate the nephrotoxicity (21, 22). In addition, furosemide causes edema of the stria vascularis, disrupts the blood-ear barrier, and enhances the entry of ototoxic drugs into the inner ear, known to potentiate cisplatin-induced hearing loss (23–25). Therefore, many researchers have sought less toxic methods for administering cisplatin without furosemide (26, 27). The second most frequent DDI detected in our study was pemetrexed and NSAIDs, mainly found in patients with lung adenocarcinoma with bone metastasis or pain. Severe hematologic toxicities in patients receiving carboplatin-based pemetrexed may be significantly induced by the inhibition of renal tubular secretion of pemetrexed through drug-drug interactions between NSAIDs and pemetrexed (28). Renal dysfunction may easily develop as a result of continued pemetrexed administration combined with NSAID therapy (29). Therefore, it is necessary to take precautions against adverse side effects when combining pemetrexed with NSAID therapy. Erlotinib was most likely to interact with various non-anticancer drugs including PPI, rifampicin, warfarin, and carbamazepine. Erlotinib is a tyrosine kinase inhibitor, which drug interactions occur commonly because of DDIs concern absorption (incomplete drug absorption is a risk of drug interaction) and metabolization by the cytochrome P450 isozymes (30). Significant clinical consequences have been associated with these interaction mechanisms (8, 31, 32). Interaction between sunitinib and ondansetron or quinolones was found in the study which may result in an increased risk of QT interval prolongation. Similar risk of interaction involved sorafenib and ondansetron. Medical oncologists should be better known about the risk of increased tyrosine kinase inhibitor toxicity or decreased tyrosine kinase inhibitor efficacy in patients given tyrosine-kinase inhibitors. If possible, the combination of some tyrosine-kinase in-hibitors and proton-pump inhibitor should be avoided. Dose adjustments of tyrosine-kinase inhibitors are highly recommended when combined with strong CYP3A4 inhibitors or inducers which can significantly affect the exposure to tyrosine-kinase inhibitors. Unless absolutely necessary, coadministration QTc- prolonging tyrosinekinase inhibitors and drugs that prolong the QTc interval should be avoided (30).

Our study revealed that patients taking more medications, those with stage IV and lung cancer patients were at greater risk of drug interactions. Patients with lung cancer were almost 5 times more likely to be exposed to drug interactions than patients with gastric cancer. The finding of Holland researchers was similar to ours. Lung cancer patients have a high risk of drug-drug interactions (33). Cisplatin, TKI, and pemetrexed which often used by lung cancer patients have a high number of DDIs. Conversely, compared with gastric cancer patients, breast and intestinal patients were only 0.73 and 0.05 times to be exposed to drug interactions. The result suggested breast and intestinal patients were at lower risk of drug interactions.

Similar to other studies (9, 34), the increasing number of medications was associated with more potential drug interactions in our study. Cancer patients with co-morbid illnesses or cancer-associated syndromes usually take more medications with potential drug-drug interactions. Patients with stage IV were also at increased risk of drug interactions because they face more treatment including disease-directed therapy, symptom management, and attention to quality of life compared with prior to stage IV.

However, different from previous studies (9, 10, 35, 36), older patients were not found to have an increased risk of DDI exposure in our study. Patients receiving chemotherapy usually need good or moderate performance status, no matter the older or the younger patients.

The present study has several limitations. First, analysis data was only from one institution, so the result might be vulnerable to institution bias. Second, many patients were administered oral therapy such as tyrosine kinase inhibitors outside the hospital, which resulted in drug-drug interactions in patients receiving tyrosine kinase inhibitors were underestimated. And the data were not analyzed to compare the characteristic differences in antineoplastic category between patients with and without DDIs due to difficult evaluation, because many patients were administered simultaneously traditional anti-tumor agents and molecule-targeted agents. In addition, documentation evidence of majority of DDIs (86.21%) was classified as fair, suggesting the interactions need more available documentation and well-controlled studies.

## Conclusion

Potential drug-drug interactions between antineoplastic drugs and non-antineoplastic drugs occur frequently in cancer patients of Chinese hospitals. Doctors should fully consider potential risk associated with DDIs. Further research should be performed to evaluate real clinical significance of these DDIs.

## Ethical considerations

Ethical issues (Including plagiarism, informed consent, misconduct, data fabrication and/or falsification, double publication and/or submission, redundancy, etc.) had been completely observed by the authors.
